# The Effects of an Oil and Wheat Flour Fortification Program on Pre-School Children and Women of Reproductive Age Living in Côte d’Ivoire, a Malaria-Endemic Area

**DOI:** 10.3390/nu8030148

**Published:** 2016-03-07

**Authors:** Fabian Rohner, Giovanna Raso, Sassor Odile P. Aké-Tano, Andreas B. Tschannen, Christopher Guy Nicholas Mascie-Taylor, Christine A. Northrop-Clewes

**Affiliations:** 1 GroundWork LLC, 40 b Les Landes, Crans-près-Céligny 1299, Switzerland; 2 Global Alliance for Improved Nutrition (GAIN), PO Box 55, Geneva 1211, Switzerland; christinaclewes@btinternet.com; 3 Centre Suisse des Recherches Scientifiques en Côte d’Ivoire, PO Box 1303, Abidjan, Côte d’Ivoire; giovanna.raso@unibas.ch (G.R.); andres.tschannen@gmail.com (A.B.T.); 4 Swiss Tropical and Public Health Institute, PO Box, Basel 4002, Switzerland; 5 University of Basel, PO Box, Basel 4003, Switzerland; 6 Institut National de Santé Publique, PO Box V 47, Abidjan, Côte d’Ivoire; odileake@yahoo.fr; 7 UFR des Sciences Médicales, Université Félix Houphouet-Boigny, PO Box V 34, Abidjan, Côte d’Ivoire; 8 Department of Biological Anthropology, University of Cambridge, Pembroke Street, Cambridge CB2 3QG, UK; nmt1@cam.ac.uk

**Keywords:** flour, oil, fortification, children, women, iron, vitamin A, anemia

## Abstract

Anemia and micronutrient deficiencies are widespread in sub-Saharan Africa, but the impact of food fortification is still debated. The objective of this study was to estimate the iron and vitamin A status of preschool children (PSC) and women of reproductive age (WRA) in households consuming fortified oil and wheat flour. The survey was cross-sectional in a rural and an urban area. Data on demographics, socioeconomic status, and fortified foods were collected at households. Hemoglobin (Hb), retinol binding protein (RBP), ferritin, soluble transferrin receptors (sTfR), subclinical inflammation, and *Plasmodium spp.* infection data were collected. In PSC, vitamin A deficiency (VAD) was prevalent, but for each 1 mg retinol equivalents (RE)/kg of oil consumed, RBP increased by 0.37 μmol/L (*p* = 0.03). In WRA, there was no significant VAD in the population (0.7%). Anemia was found in 92.2% of rural and 56.3% of urban PSC (*p* < 0.001). PSC with access to adequately fortified flour had Hb concentrations 15.7 g/L higher than those who did not (*p* < 0.001). Hb levels increased by +0.238 g/L per mg/kg increase in iron fortification levels (*p* < 0.001). The national program fortifying vegetable oil with vitamin A and wheat flour with iron and folic acid may have contributed to improved micronutrient status of PSC from two areas in Côte d’Ivoire.

## 1. Introduction

Anemia is one of the most widespread public health problems affecting communities in many countries. The primary cause is thought to be iron deficiency, but other factors, such as inflammation, infections, parasites, nutritional deficiencies, and hemoglobinopathies, have a role to play in the etiology [1]. In Côte d’Ivoire, the Ministry of Health (MoH) adopted multiple strategies to combat anemia, including iron supplementation, dietary counseling to encourage food diversification, food fortification, and public health measures to aid in the reduction of malaria and other parasitic diseases. One strategy to fight iron deficiency anemia (IDA) in pregnant women is supplementation with iron and folic acid tablets given during prenatal and postpartum consultations [2].

Vitamin A deficiency (VAD) is a major nutritional problem in many lower-income countries, where poor diets and exposure to inflammation coexist [3]. Efforts to reduce VAD in Côte d’Ivoire include the administration of vitamin A capsules to preschool children (PSC) 6–59 months, in conjunction with routine immunization programs, e.g., the Expanded Program on Immunization (EPI)-Plus [4].

As a complement to the supplementation programs, the Global Alliance for Improved Nutrition (GAIN) supported the Ivorian program for promotion of fortified food in Côte d’Ivoire—Programme Ivoirien de la Promotion des Aliments Fortifiés [PIPAF])—to fortify wheat flour with electrolytic iron and folic acid and to fortify palm oil with vitamin A. Under PIPAF, two laws pertaining to the fortification of vegetable oil and wheat flour were passed in 2007, rendering the fortification of these two vehicles mandatory. Fortified oil was on the market prior to PIPAF, and it was estimated that 55% of the commercialized vegetable oil was fortified at that time (unpublished data). PIPAF aimed to increase the coverage to 80% over a period of 3 years. No wheat flour was fortified prior to program launch in 2007.

Before the introduction of PIPAF, a national micronutrient survey was conducted in Côte d’Ivoire in 2007 [5] to provide an update on the prevalence of anemia, iron deficiency (defined as low serum ferritin concentrations), and VAD among PSC and women of reproductive age (WRA) (15–49 years) and on the prevalence of folate and vitamin B_12_ deficiency among WRA. The survey was designed to provide national estimates and covered a total of 900 households in 9 eco-regions of Côte d’Ivoire. Anemia prevalence was high in both surveyed populations (WRA: 50%, PSC: 72%), whereas the prevalence of VAD among WRA (1%) and iron deficiency among both PSC (16%) and WRA (17%) were surprisingly low. The prevalence of VAD was high among PSC (24%). Folate deficiency among WRA was universally high (86%), and vitamin B_12_ deficiency (measured in only a subsample of WRA) was 18% [5].

This paper presents data from a cross-sectional survey conducted in 2010 that aimed to identify associations between the consumption of fortified wheat flour with electrolytic iron and folic acid and of oil with vitamin A and the iron and vitamin A status of PSC and WRA in two target populations in Côte d’Ivoire, one living in a rural setting and one living in an urban setting, in households consuming wheat flour and/or oil.

## 2. Methods

### 2.1. Study Design

The survey was cross-sectional and the data collected were used to evaluate the micronutrient status of PSC and WRA consuming flour and oil. The survey took place in October 2010, and followed the last vitamin A supplementation round (May 2010) by at least 4 months and preceded the next round of supplementation, expected in November 2010 (but which did not take place due to the outbreak of the civil war around that period).

The sampling area was representative of the rural part of the Bouaflé District located in central Côte d’Ivoire and Abidjan, by selecting at random three communes in the urban areas of Abidjan in the southern part of the country: Treichville, Attécoubé, and Abobo. A total of 26 clusters for the urban area and 26 clusters for the rural area were selected independently by a two-stage process (probability proportional to population size and random selection), leading to a total of 52 clusters for both urban and rural strata. In each cluster, 15 households were selected using the EPI method [4]. All PSC (aged 6–59 months) and WRA (aged 15–49 years) of a selected household were enrolled.

The eligibility criteria for respondents were: to be of correct age/sex, not pregnant (WRA; self-reported), consenting to take part in the study, and without any known counter indications for blood sampling (e.g., known hemophilia).

### 2.2. Enrollment of Participants, Food Sampling and Analysis

After household selection and consent to take part in the survey, all consenting and eligible subjects were registered (household, sex, age, date, ID number). In interview sessions, the respondents answered questions on demographics, health, and socioeconomic status (SES; housing quality, access to water, electricity, transport), as well as on frequency and quantity of purchases of oil and wheat flour products. Subsequently, eligible household members were invited to go to the nearest health facility to give a blood sample.

During the interview, respondents were asked whether they could provide a small sample of wheat flour and vegetable oil. If an oil or flour sample could not be collected from the interviewee, she was asked where the oil and flour used by the household were normally purchased, and, if possible, she described the brand and packaging. Finally, the interviewee was asked to accompany a member of the team to the shop to purchase the correct oil or flour for analysis.

After collection at the household or in the nearby shop, oil samples were stored in boxes in a dark and cool location before being analyzed in-country using a rapid method (iCheck™-iEx [6]). Flour samples, kept dry and cool, were shipped to the Swiss Vitamin Institute (Epalinges, Switzerland) upon completion of the fieldwork, where they were analyzed for iron content. The iron content in the wheat flour was quantified using atomic absorption spectrometry after dry ashing [7]. The laboratory takes part in regular inter-laboratory testing.

### 2.3. Blood Sampling and Analysis

Venous blood samples were collected from antecubital veins of WRA and children at least 12 months old; younger children were sampled from the heel. The venous blood samples (4 mL) were drawn into ethylenediaminetetraacetic acid (EDTA)-treated evacuated tubes (Vacutainer^®^, Becton Dickinson, Franklin Lakes, NJ, USA). Immediately after blood sampling, hemoglobin (Hb) concentration was determined using a Hemocue^®^ device (Hemocue, Angelsborg, Sweden), and results were noted and given to the respondent. For quality control, liquid reference samples provided by the supplier were used daily. On diagnosis of severe anemia (Hb concentration < 70 g/L), the field team conducted a rapid malaria test (SD Bioline Malaria Antigen™, Standard Diagnostics Inc., Yongin-Si, Korea). Respondents who were diagnosed with malaria were treated with an artemisinin-based combination therapy (ACT) (*i.e.*, artesunate-amodiaquine (Co-arinate), Dafra Pharma, Turnhout, Belgium). ACTs have recently been adapted as the first-line malaria treatment in Côte d’Ivoire [7]. If malaria testing was negative, an iron supplementation course, free of charge, was provided according to national guidelines.

After phlebotomy and on-site diagnostics, the remaining whole blood was stored on ice and protected from direct light until further processing. Later the same day, thick and thin blood films for malaria testing were prepared, stained with Giemsa, and dried for storage. Subsequently, the remaining whole blood was centrifuged and the plasma aliquoted and frozen. The plasma was later transported while frozen, and stored at −25 °C for later analysis.

Malaria slides were examined under a microscope for species-specific *Plasmodium* infection. Parasites were counted against 200 leukocytes (if <10 parasites were identified, counting was continued up to 500 leukocytes). Counts were converted to the number of parasites/μL of blood, assuming a leukocyte count of 8000/μL [8].

Plasma retinol binding protein (RBP), ferritin, soluble transferrin receptors (sTfR), C-reactive protein (CRP), and α-1-acid-glycoprotein (AGP) were measured at the VitMin laboratories (Willstaett, Germany) using the sandwich enzyme-linked immunosorbent assay (ELISA) method of Erhardt *et al.* [9]. The laboratory is a participant in the U.S. Centers for Disease Control and Prevention (CDC) Vitamin A Laboratory-External Quality Assurance (VITAL-EQA) inter-laboratory comparison rounds and has a rigorous internal quality control system.

### 2.4. Ethics and Consent

Approval for the study was granted by the ethical committee of the MoH in Côte d’Ivoire (Comité National d’Ethique et de la Recherche, number 5713/2010/MSHP/CNER). Inclusion in the survey was dependent on the household head being willing to participate in the survey and giving written informed consent for phlebotomy. Furthermore, written informed consent was sought from adult female participants, as well as from parents or legal guardians of participating children.

### 2.5. Data Management and Statistical Analysis

All field data were double-entered and cross-checked using EpiData Entry version 3.1 Laboratory data were either auto-generated or double-entered (Microsoft Excel, version 97–2003).

Statistical analysis was conducted using SPSS version 20 (IBM corporation, Armonk, NY, USA). Continuous data were checked for skewness using the Cox test (coefficient of skewness divided by the standard error of skewness), as well as by examination of the frequency distribution. The relationship between two categorical variables was analyzed by the chi-square test and between continuous variables by independent-sample *t*-test or one-way analysis of variance (ANOVA). Curve estimations included testing for linear and quadratic effects of the continuous independent variables. Sequential multiple regression analyses were used to analyze dependent continuous variables with two or more independent variables, and binary and multinomial logistic regression analyses were used to predict group membership based on categorical and continuous independent variables.

### 2.6. Thresholds for Blood Parameters

A Hb concentration of <110 g/L in children 6–59 months old and a concentration of <120 g/L in WRA are the World Health Organization (WHO) cutoffs for anemia [10]. In populations, anemia prevalence of ≥40% indicates a severe public health problem, 20.0%–39.9% a moderate, and 5.0%–19.9% a mild; where the prevalence is ≤4.9%, the population is described as normal [10]. Plasma sTfR concentrations of >8.3 mg/L indicate iron deficient erythropoiesis [9]. Iron deficiency (depleted iron stores) was defined by low ferritin concentrations. The WHO cutoff is <12 µg/L for children <5 years and <15 µg/L for everyone ≥5 years, but it was adjusted according to inflammatory status (see next paragraph) [11]. IDA was defined as having anemia and depleted iron stores. VAD was defined as RBP concentrations <0.7 µmol/L, assuming equivalence to serum retinol [12]. The acute phase proteins, CRP and AGP, were used to classify inflammation into four categories [13]: (1) normal or no inflammation—no elevated acute phase proteins; (2) incubation period defined as a CRP concentration of >5 mg/L; (3) early convalescence defined as a CRP concentration >5 mg/L and an AGP concentration >1 g/L; and (4) late convalescence defined as an elevated AGP concentration >1 g/L.

Ferritin concentrations are increased by inflammation, even in apparently healthy people; hence the prevalence of iron deficiency may be underestimated in populations where inflammation is common. WHO recommends serum ferritin concentrations as the best indicator of depleted iron stores, a precondition for iron deficiency, in the absence of inflammation [1]. Given the presence of inflammation, ferritin concentrations were corrected using inflammation markers according to the meta-analysis of Thurnham *et al.* [14].

Retinol concentrations are reduced by the presence of inflammation; hence the prevalence of VAD can be overestimated. A similar adjustment to that described above can also be done for retinol concentrations [13]. In this study, RBP, not retinol, concentrations were measured, but there was an excellent correlation between the retinol and RBP concentrations in the CDC VITAL-EQA program carried out at the time of this analysis. Therefore, the correction for inflammation proposed for serum retinol was applied to adjust the RBP.

### 2.7. Calculation of Daily Vitamin A Intake from Oil

The calculation of the daily vitamin A intake from oil was done as follows: (1) daily quantity of oil consumed at the household was calculated from the money spent on purchasing oil (using price/L) and the reported frequency of purchase; the amount of oil in liters was then converted to oil in kg (using the conversion factor of 0.8875 g/mL at 25 °C) [15]; (2) the amount of vitamin A consumed per household was obtained using the measured concentration of vitamin A in mg RE/kg multiplied by the amount of oil (in kg) consumed on a daily basis; and (3) the number of “consumption unit equivalents” was then calculated according to Gibson [16]. For this, the number of household members in each age group was calculated, and the number of “consumption units” summed together, enabling the calculation of oil consumption per individual household member.

## 3. Results

### 3.1. Food Samples: Oil Vitamin A Content

The legally mandated level for the fortification of oil with vitamin A in Côte d’Ivoire is 8 μg/g RE. Overall, 32.2% of oil samples were fortified at the legal level or above, and there was no significant difference in the coverage between the rural area of Bouaflé (33.5%) and the urban area of Abidjan (30.8%). Although 8 μg/g RE is the legal level of fortification at the refinery, at the household, variation in the level of fortification of about 20% was deemed acceptable. Therefore, any oil containing ≥6.4 μg/g RE was considered adequately fortified. More than 50% of the oil was in this category, with no rural/urban differences. Concentrations of ≤3.2 μg/g RE in oil were considered fortified at unacceptable levels, and 35% of urban and 41% of rural samples fell into this category. Any oil that had levels >3.2 μg/g RE but <6.4 μg/g RE was considered inadequately fortified; there were significant urban-rural differences in this category (14.2% *vs.* 9.2%, respectively; *p* < 0.03).

### 3.2. Flour Iron Content

The legal level for the fortification of flour with electrolytic iron is 60 mg/kg. Overall, 69.1% of flour samples were fortified to the legal level or above, and there was a significant difference in the prevalence of adequately fortified flour between the rural area of Bouaflé (47%) and the urban area of Abidjan (93%; *p* < 0.001). Some fluctuation during the fortification process of flour is normal, which means that any flour containing ≥48 mg/kg of iron was considered adequately fortified at the household level, and 100% of urban flour samples were in this category, but only 50% of rural samples. A third of rural samples were considered “unfortified” with concentrations of iron ≤ 24 mg/kg, but hardly any samples from the urban stratum fell into this category (<1%; *p* < 0.001). Any flour that had iron levels >24 mg/kg but <48 mg/kg was considered inadequately fortified.

### 3.3. Inflammation and Plasmodium Spp. Parasites

**PSC:** Overall, 49.8% and 37.2% of PSC had elevated AGP and CRP concentrations, respectively, and there was a significant difference between rural and urban populations (AGP: 69.7% rural, 27.8% urban, *p* < 0.001; CRP 53.3% rural, 18.5% urban, *p* < 0.001). *Plasmodium* parasites were found in 29.1% of PSC, with significant differences by residency (45.8% rural, 7.5% urban; *p* < 0.001).

**WRA:** There was far less inflammation in WRA, as only 10.3% had elevated AGP concentrations and 13.2% had elevated CRP concentrations, without any urban/rural differences. *Plasmodium* parasites were found in 9.9% of WRA, and there were significant rural/urban differences (13.0% rural, 7.3% urban; *p* < 0.008).

### 3.4. Vitamin A Status

**PSC:** The inflammation-adjusted overall mean RBP concentration was 0.94 μmol/L, with boys having a lower mean value (0.92 μmol/L) than girls (0.96 μmol/L) (*p* < 0.05). The RBP concentration was 0.13 μmol/L lower in rural areas than in urban areas. Males and females in the urban setting had significantly less VAD than those living in rural areas (males: urban 13.2%, rural 24.4%, *p* < 0.001; females: urban 6.5%, rural 24.3%, *p* < 0.04), but there was no significant difference in the prevalence of adjusted VAD between sexes (19.3% and 16.5%, males and females, respectively). There was no relationship between RBP concentrations and age of the child. Concentrations of RBP in PSC with *Plasmodium* parasites (0.85 µmol/L) were significantly lower than those without parasites (0.99 µmol/L) (*p* < 0.001), and, after correcting for age, sex, and residency, there was still a significant residual difference (−0.09 µmol/L) (*p* < 0.001). Overall, more than 15% of the children in the population surveyed had VAD, which, according to the WHO, suggests a public health problem among that age group [14], assuming retinol and RBP cutoffs are equivalent.

**WRA:** Among WRA, there was no significant difference in mean RBP or differences in VAD by residency, or in the presence of *Plasmodium* parasites; nor was there any significant age effect. The mean adjusted RBP concentration was 1.64 μmol/L, and there was no significant VAD (0.7%) in the population using adjusted concentrations.

### 3.5. Iron Status

**PSC:** The mean Hb concentration was significantly higher in urban than in rural children (Table 1). The majority of rural children (92.2%; *p* < 0.001) were anemic (Hb < 110 g/L) compared with just over half of the urban children (56.3%; *p* < 0.001), and nearly 1 in 5 rural children had severe anemia (Hb concentration < 70 g/L) compared to only 1 in 38 of urban children (Table 1). Hb concentration increased significantly with age (0.278 g/L/month), and a stepwise binary logistic regression analysis revealed that, after removing the effects of the age and sex of the child, residency remained significant for Hb concentration.

The acute phase proteins CRP and AGP were used to correct the effect of inflammation on ferritin concentrations [14]. Adjusted mean ferritin concentrations (logged and unlogged) were significantly higher in rural than urban children (Table 1). The percentage of those with iron deficiency (defined as ferritin concentrations <12 μg/L) was small, but was significantly higher in urban than rural children (Table 1). The presence of *Plasmodium* parasites increased logged ferritin concentrations (62.9 µg/L) compared to those not infected (42.5 µg/L) (*p* < 0.001), and, after correction for age, sex, and residency, there was still a residual difference between those with and those without parasites (+12 µg/L; *p* < 0.001). Adjusted ferritin concentrations increased significantly with age (0.374 μg/L/month), and stepwise binary logistic regression analyses revealed that, after removing the effects of the age and sex of the child, residency remained significant for ferritin concentrations.

By residency, prevalence of iron deficient erythropoiesis (elevated sTfR values) was low and there were no significant differences. The mean sTfR concentration in children in the rural area was greater than the upper level of the accepted normal range (>8.3 mg/L), and there were significant differences between urban and rural children (Table 1). sTfR concentrations were higher in those with *Plasmodium* parasites (11.2 mg/L) than in those without (6.4 mg/L) (*p* < 0.001), and, after correction for age, sex, and residency, a significant difference remained (+2.6 mg/L; *p* < 0.001). sTfR concentrations fell with increase in age, on average by 0.061 mg/L/month, and stepwise binary logistic regression analyses revealed that, after removing the effects of the age and sex of the child, residency no longer remained significant for sTfR concentrations.

**WRA:** Mean Hb concentrations were significantly higher among urban than rural WRA, while the opposite was found for concentrations of sTfR. There were no significant differences in mean ferritin by residency (Table 2). More than three-quarters of rural WRA were anemic compared to less than half of the urban sample (Table 2). There were no significant differences in the prevalence of iron deficiency (unlogged or logged) by residency, and the presence of *Plasmodium* parasites had no significant effect on sTfR concentrations.

Hb concentrations fell by, on average, 0.32 g/L/year of age among WRA, while ferritin concentrations increased, on average, by 6.4 μg/L/year of age. Stepwise binary logistic regression analyses revealed that, after removing the effects of age, residency remained significant for Hb concentration.

### 3.6. Relationship between Fortified Oil Intake and Vitamin A Status

There was a significant positive relationship between RBP concentrations and vitamin A consumption from the oil in PSC (*p* = 0.03), so that for each 1 mg RE/kg increase in vitamin A consumption, RBP increased, on average, by 0.37 μmol/L. Dividing the PSC into two age groups, those 6.0–23.9 months and those 24–59 months, the relationship between RBP concentrations and vitamin A consumption from the oil was no longer significant in the younger age group (RBP increased by 0.26 μmol/L per unit oil). In those 24–59 months, there was a significant relationship, since, for each 1 mg RE/kg increase in vitamin A consumption, RBP increased by 0.58 μmol/L (*p* = 0.004). When analyzed separately in urban and rural localities, the relationship was still positive, but not significant. When an interaction effect was added to the model it was not statistically significant.

Among WRA, there were no associations found with prevalence or mean RBP concentrations, but there was very little VAD to start with.

### 3.7. Relationship between Flour Fortification Levels and Anemia

A sequential multiple regression analysis was undertaken, removing the effects of age and sex, to test whether the three categories of iron fortification had an effect on Hb concentration in PSC. Those PSC having access to flour with <24 mg/kg iron had Hb concentrations 14.3 g/L lower and those having access to flour with ≥24 to ≤48 mg/kg iron had Hb concentrations 15.7 g/L lower than those in the group having access to flour with ≥48 mg/kg iron (post hoc test results which show that both group means are *p* < 0.001 lower than the ≥48 mg/kg group mean). This association was slightly less strong but remained significant after correction for the presence of *Plasmodium* parasites (−11.7 g/L and −10.7 g/L, respectively; both *p* < 0.001 compared with ≥48 mg/kg group mean) (Table 3). Repeating the regression model, but using only children who were negative for *Plasmodium* parasites, the effects of age and sex were removed, but the association between fortification category and mean Hb remained highly significant: children having access to flour with <24 mg/kg iron had Hb concentrations 15.1 g/L lower and those having access to flour with ≥24 to ≤48 mg/kg iron had Hb concentrations 16.2 g/L lower than those having access to flour with ≥48 mg/kg iron (*post hoc* test results which show that both group means are *p* < 0.001 lower than the ≥48 mg/kg group mean) (Table 3). In all these analyses there were no significant differences between <24 mg/kg and ≥24–≤48 mg/kg group means and the *p* value shown in Table 3 refers to the overall comparison of the three means in each analysis. The analyses were repeated using iron fortification levels as a continuous variable. With all children in the model and after removing the effects of age and sex, Hb increased by +0.238 g/L for each unit (mg/kg) increase in iron (*p* < 0.001). Repeating the analysis using only *Plasmodium*-free children in the model, the increase in Hb was +0.254 g/L per unit of iron (*p* < 0.001).

## 4. Discussion

The cross-sectional data presented estimates of the coverage of the fortified flour and oil and the impact of consuming fortified oil on the vitamin A status of PSC and WRA and of consuming fortified flour on the iron status of PSC and WRA, 3 years after the introduction of a national fortification program (PIPAF). The study was not designed to be nationally representative; rather, two regions were selected that showed a heterogeneous coverage in a previous food fortification coverage survey (unpublished data). Because of the design of the survey, using only one rural and one urban area, it was not possible to estimate the impact of the PIPAF program by comparison between the national survey done in 2007 and the present survey. Further, as briefly described in the introduction, important socioeconomic and political changes between these two surveys are further weakening comparability of the two datasets [5].

In this study, about 50% of oil samples collected were adequately fortified. Iron fortification was less consistent, as almost 100% of the flour samples in the urban stratum were found to have adequate iron content, while coverage was only about 50% in the rural area. This study was not designed to find explanations for these findings, but due to the centralized production of wheat flour, they are somewhat unexpected.

In the PSC, VAD was still of public health concern, but the prevalence was improved in Bouaflé and Abidjan (17.8%) compared to the overall results (not specific to the two settings) obtained in the national survey carried out in 2007 (24%) [5]. Using the categorical definitions (*i.e.*, <6.4 or ≥6.4 µg RE/g oil), no relationship was found between the fortified oil consumed and vitamin A status in PSC or WRA. However, when the daily quantity of oil consumed was calculated for each population group, a significant positive relationship was found between RBP concentration and vitamin A consumption in the PSC, an important finding potentially showing a positive impact of the oil fortification program on plasma vitamin A concentrations. It is noteworthy that the study design does not allow inferring causation but is limited to demonstrating significant associations. No relationship was found in the WRA, but VAD prevalence was very low in this population group, and a relationship was thus not expected.

The findings show that the methodological aspects of evaluating the effect of fortification programs are very important in population-based studies, which aim to demonstrate associations between micronutrient intake and status. Status should ideally be linked with intake estimates, but one of the limitations of this survey was that it was not possible to do intake calculation for flour because the population does not consume flour, but rather processed foods made from flour, such as bread or dumplings. Quantification would have been possible only if weighed food intake records were undertaken, which was not feasible during the survey. However, the study did find a significant association between Hb levels and the iron content of the flour, which was also biologically relevant to PSC. Hb concentrations were at least 14 g/L higher in children in households with adequately fortified flour, regardless of whether they had *Plasmodium* parasitemia or not. Including only *Plasmodium*-free children in the analyses produced an even larger difference, indicating that the presence of the parasites had a masking or negative effect on the association between the fortification and Hb concentrations.

Data from this study indicate that *Plasmodium* parasites were associated with higher plasma levels of the acute phase proteins, ferritin, and sTfR and lower concentrations of Hb and RBP, which has implications for the interpretation of the biological impact of the fortification program. Concentrations of ferritin and RBP, after adjustment for inflammation, showed residual differences of +12 µg/L and −0.09 µmol/L, respectively, between those with and those without *Plasmodium* parasites, indicating that the meta-analysis correction for inflammation may not completely adjust concentrations for malaria, as indicated by presence of *Plasmodium* parasites [14,17]. However, due to the complexities of interpreting the iron status biomarkers (ferritin and sTfR), we focused our analysis on the biological endpoint: Hb concentration. It is well-known that many trials of iron fortification in malaria-endemic areas of Africa have been ineffective [18,19] or have had only limited effect on anemia [20,21]. Malaria may induce iron deficiency through reduced iron absorption or iron loss after hemolysis, as well as by sequestration of iron in macrophages of the mononuclear phagocyte system. Although the subjects in this survey were apparently healthy at the time of the blood sampling, those with *Plasmodium* parasites were already producing an acute phase response stimulated by an increase in cytokine concentrations, such as interleukin-6 [17,22], which also stimulate hepatic hepcidin production. High circulating hepcidin concentrations reduce iron absorption from the gut by blocking the iron-transporter ferroportin and can increase iron sequestration in the reticuloendothelial system [23]. The resulting hypoferremia limits the iron available for erythropoiesis and contributes to anemia. A study by Cercamondi *et al.* [24] confirmed this hypothesis, as they showed that afebrile malarial parasitemia decreased dietary iron absorption. The effect appears to be due to the low-level inflammation modulation of serum hepcidin and may help explain why the iron fortification of flour, used in these two populations in Côte d’Ivoire, may be less effective in those with *Plasmodium* parasites.

## 5. Conclusions

The data from this cross-sectional survey indicate that a national program fortifying vegetable oil with vitamin A and wheat flour with iron and folic acid may have contributed to improving the micronutrient status of PSC from an urban area and a rural area in Côte d’Ivoire. Most importantly, lower VAD prevalence was found in those consuming adequately fortified oil, and higher Hb concentrations were found in those children having access to adequately fortified flour products.

## Figures and Tables

**Table 1:** Mean Hb, ferritin, and sTfR concentrations and prevalence of anemia, iron deficiency, and iron deficient erythropoiesis in PSC by residency.

Variable		Urban				Rural			*p*		Total Population		
	*N*	Arithmetic Mean	Geometric Mean	%	*N*	Arithmetic Mean	Geometric Mean	%		*N*	Arithmetic Mean	Geometric Mean	%
Hb g/L	348	104.6	–	–	387	85.0	–	–	<0.001	735	94.3	–	–
Hb < 70 g/L	348	–	–	2.6	387	–	–	17.8	<0.001	735	–	–	10.6
Hb ≥ 70, <110 g/L	348	–	–	53.7	387	–	–	74.4	<0.001	735	–	–	64.6
Hb ≥ 110 g/L	348	–	–	43.7	387	–	–	7.8	<0.001	735	–	–	24.8
Ferritin μg/L ^a^	335	38.3	29.3	–	390	59.5	45.3	–	<0.001	725	49.7	34.7	–
Ferritin < 12 µg/L ^a^	335	–	–	22.1	390	–	–	6.9	<0.001	725	–	–	13.9
sTfR mg/L	335	5.1	4.6	–	390	10.6	9.0	–	<0.001	725	8.1	6.3	–
sTfR > 8.3 mg/L	335	–	–	10.7	390	–	–	8.7	ns ^b^	725	–	–	9.7

^a^ Ferritin adjusted according to [14]; ^b^ Not significant.

**Table 2:** Mean Hb, ferritin, and sTfR concentrations and anemia, depleted iron stores, and iron deficiency among WRA, by residency.

Variable		Urban				Rural			*p*		Total Population		
	*N*	Arithmetic Mean	Geometric Mean	%	*N*	Arithmetic Mean	Geometric Mean	%		*N*	Arithmetic Mean	Geometric Mean	%
Hb g/L	379	119.6	–	–	309	105.3	–	–	<0.001	688	113.2	–	–
Hb < 70 g/L	379	–	–	0.5	309	–	–	4.2	<0.001	688	–	–	2.2
Hb ≥ 70, <120 g/L	379	–	–	44.1	309	–	–	73.5	<0.001	688	–	–	73.5
Hb ≥ 120 g/L	379	–	–	55.4	309	–	–	22.3	<0.001	688	–	–	22.3
Ferritin μg/L ^a^	378	58.3	41.0	–	314	56.5	40.4	–	ns ^b^	692	57.5	40.7	–
Ferritin < 12 µg/L ^a^	378	–	–	10.1	314	–	–	9.6	ns ^b^	692	–	–	9.8
sTfR mg/L	378	4.8	4.5	–	314	7.0	6.5	–	<0.001	692	5.8	5.3	–
sTfR > 8.3 mg/L	378	–	–	6.3	314	–	–	9.6	ns ^b^	692	–	–	7.8

^a^ Ferritin adjusted according to [14]; ^b^ Not significant.

**Table 3:** Differences in Hb concentrations according to the fortification level of iron in wheat flour among PSC.

Category of Iron Fortification of Flour	Difference in Hb (g/L)		Difference in Hb (g/L) in Children without Malaria
	After Correction for Age and Sex	After Correction for Age, Sex, and *Plasmodium*	After Correction for Age and Sex
<24 mg/kg	−14.3	−11.7	−15.1
≥24–≤48 mg/kg	−15.7	−10.7	−16.2
≥48 mg/kg	– ^a^	– ^a^	– ^a^
*p*	<0.001	<0.001	<0.001

^a^ Reference mean concentration.

## References

[1] WHO/CDC (2007). Assessing the Iron Status of Populations, Including Literature Reviews: Report of a Joint World Health Organization/Centers for Disease Control and Prevention Technical Consultation on the Assessment of Iron Status at the Population Level (6–8 April 2004).

[2] WHO Weekly Iron-Folic Acid Supplementation (WIFS) in Women of Reproductive Age: Its Role in Promoting Optimal Maternal and Child Health. Position Statement, 2009. http://www.who.int/nutrition/publications/micronutrients/weekly_iron_folicacid.pdf.

[3] WHO (2009). Global Prevalence of Vitamin A Deficiency in Populations at Risk, 1995–2005. WHO Global Database on Vitamin A Deficiency.

[4] WHO (2007). The EPI Coverage Survey—Training for Mid-Level Managers (WHO/IVB/08.07).

[5] Rohner F., Northrop-Clewes C., Tschannen A.B., Bosso P.E., Kouassi-Gohou V., Erhardt J.G., Bui M., Zimmermann M.B., Mascie-Taylor C.G. (2014). Prevalence and public health relevance of micronutrient deficiencies and undernutrition in pre-school children and women of reproductive age in cote d’ivoire, west africa. Public Health Nutr..

[6] Rohner F., Frey S.K., Mothes R., Hurtienne A., Hartong S., Bosso P.E., Bui M., Schweigert F.J., Northrop-Clewes C.A. (2011). Quantification of vitamin a in palm oil using a fast and simple portable device: Method validation and comparison to high-performance liquid chromatography. Int. J. Vitam. Nutr. Res..

[7] WHO Malaria Control Today—Current WHO Recommendations, Working Document. http://www.who.int/malaria/publications/mct_workingpaper.pdf.

[8] WHO (1990). Diagnosis of Malaria.

[9] Erhardt J.G., Estes J.E., Pfeiffer C.M., Biesalski H.K., Craft N.E. (2004). Combined measurement of ferritin, soluble transferrin receptor, retinol binding protein, and C-reactive protein by an inexpensive, sensitive, and simple sandwich enzyme-linked immunosorbent assay technique. J. Nutr..

[10] WHO/UNICEF/UNU (2001). Iron Deficiency Anaemia. Assessment, Prevention and Control. A Guide for Programme Managers.

[11] WHO Serum Ferritin Concentrations for the Assessment of Iron Status and Iron Deficiency in Populations. Vitamin and Mineral Nutrition Information System, 2011. http://www.who.int/vmnis/indicators/serum_ferritin.pdf.

[12] WHO (1996). Indicators for Assessing Vitamin A Deficiency and Their Application in Monitoring and Evaluating Intervention Programmes.

[13] Thurnham D.I., McCabe G.P., Northrop-Clewes C.A., Nestel P. (2003). Effects of subclinical infection on plasma retinol concentrations and assessment of prevalence of vitamin A deficiency: Meta-analysis. Lancet.

[14] Thurnham D.I., McCabe L.D., Haldar S., Wieringa F.T., Northrop-Clewes C.A., McCabe G.P. (2010). Adjusting plasma ferritin concentrations to remove the effects of subclinical inflammation in the assessment of iron deficiency: A meta-analysis. Am. J. Clin. Nutr..

[15] Chempro Top-Notch Technology in Production of Oils and Fats. http://www.chempro.in/palmoilproperties.htm.

[16] Gibson R.S. (2005). Principles of Nutritional Assessment.

[17] Wenisch C., Linnau K.F., Looaresuwan S., Rumpold H. (1999). Plasma levels of the interleukin-6 cytokine family in persons with severe Plasmodium falciparum malaria. J. Infect. Dis..

[18] Van Stuijvenberg M.E., Smuts C.M., Lombard C.J., Dhansay M.A. (2008). Fortifying brown bread with sodium iron EDTA, ferrous fumarate, or electrolytic iron does not affect iron status in South African schoolchildren. J. Nutr..

[19] Rohner F., Zimmermann M.B., Amon R.J., Vounatsou P., Tschannen A.B., N’Goran E.K., Nindjin C., Cacou M.C., Te-Bonle M.D., Aka H. (2010). In a randomized controlled trial of iron fortification, anthelmintic treatment, and intermittent preventive treatment of malaria for anemia control in Ivorian children, only anthelmintic treatment shows modest benefit. J. Nutr..

[20] Wegmuller R., Camara F., Zimmermann M.B., Adou P., Hurrell R.F. (2006). Salt dual-fortified with iodine and micronized ground ferric pyrophosphate affects iron status but not hemoglobin in children in Cote d’Ivoire. J. Nutr..

[21] Andang’O P.E., Osendarp S.J., Ayah R., West C.E., Mwaniki D.L., De Wolf C.A., Kraaijenhagen R., Kok F.J., Verhoef H. (2007). Efficacy of iron-fortified whole maize flour on iron status of schoolchildren in Kenya: A randomised controlled trial. Lancet.

[22] Lyke K.E., Burges R., Cissoko Y., Sangare L., Dao M., Diarra I., Kone A., Harley R., Plowe C.V., Doumbo O.K. (2004). Serum levels of the proinflammatory cytokines interleukin-1 beta (IL-1beta), IL-6, IL-8, IL-10, tumor necrosis factor alpha, and IL-12 (p70) in Malian children with severe Plasmodium falciparum malaria and matched uncomplicated malaria or healthy controls. Infect. Immunity.

[23] Weiss G. (2009). Iron metabolism in the anemia of chronic disease. Biochim. Biophys. Acta.

[24] Cercamondi C.I., Egli I.M., Ahouandjinou E., Dossa R., Zeder C., Salami L., Tjalsma H., Wiegerinck E., Tanno T., Hurrell R.F. (2010). Afebrile Plasmodium falciparum parasitemia decreases absorption of fortification iron but does not affect systemic iron utilization: A double stable-isotope study in young Beninese women. Am. J. Clin. Nutr..

